# The metabolic effects of intermittent versus continuous feeding in critically ill patients

**DOI:** 10.1038/s41598-023-46490-5

**Published:** 2023-11-09

**Authors:** D. Wilkinson, I. J. Gallagher, A. McNelly, D. E. Bear, N. Hart, H. E. Montgomery, A. Le Guennec, M. R. Conte, T. Francis, S. D. R. Harridge, P. J. Atherton, Z. A. Puthucheary

**Affiliations:** 1 MRC-Versus Arthritis Centre for Musculoskeletal Ageing Research, Metabolic and Molecular Physiology, University of Nottingham, Queen’s Medical Cetnre, Nottingham, UK; 2 National Institute for Health Research (NIHR) Nottingham Biomedical Research Centre, Nottinghan University Hospitals and University of Nottingham, Queen’s Medical Centre, Nottingham, UK; 3 School of Medicine, University of Nottingham, Royal Derby Hospital, Derby, UK; 4 University of Sterling, Stirling, UK; 5 William Harvey Research Institute, Barts and The London School of Medicine and Dentistry, Queen Mary University of London, London, UK; 6 Department of Nutrition and Dietetics St Thomas’ NHS Foundation Trust, London, UK; 7 Department of Critical Care, Guy’s and St. Thomas’ NHS Foundation & King’s College London (KCL) NIHR BRC, London, UK; 8 Centre for Human and Applied Physiological Science, King’s College London, London, UK; 9 Lane Fox Respiratory Service, Guy’s & St Thomas’ Foundation Trust, London, UK; 10 Lane Fox Clinical Respiratory Physiology Research Centre, Kings College London, London, UK; 11 Department of Medicine and Centre for Human Health and Performance, University College London (UCL), London, UK; 12 Centre for Biomolecular Spectroscopy, Guy’s Campus, King’s College London, London, UK; 13 Randall Centre for Cell and Molecular Biophysics, Guy’s Campus, King’s College London, London, UK; 14 Adult Critical Care Unit, Royal London Hospital, Whitechapel, London, E1 1BB UK

**Keywords:** Clinical trial design, Translational research

## Abstract

Intermittent (or bolus) feeding regimens in critically ill patients have been of increasing interest to clinicians and scientists. Changes in amino acid, fat and carbohydrate metabolites over time might yet deliver other benefits (e.g. modulation of the circadian rhythm and sleep, and impacts on ghrelin secretion, insulin resistance and autophagy). We set out to characterise these changes in metabolite concentration. The Intermittent versus Continuous Feeding in Critically Ill paitents study (NCT02358512) was an eight-centre single-blinded randomised controlled trial. Patients were randomised to received a continuous (control arm) or intermittent (6x/day, intervention arm) enteral feeding regimen. Blood samples were taken on trial days 1, 7 and 10 immediately before and 30 min after intermittent feeds, and at equivalent timepoints in the control arm. A pre-planned targeted metabolomic analysis was performend using Nuclear Resonance Spectroscopy. Five hundred and ninety four samples were analysed from 75 patients. A total of 24 amino acid-, 19 lipid based-, and 44 small molecule metabolite features. Across the main two axes of variation (40–60% and 6–8% of variance), no broad patterns distinguished between intermittent or continuous feeding arms, across intra-day sampling times or over the 10 days from initial ICU admission. Logfold decreases in abundance were seen in metabolites related to amino acids (Glutamine − 0.682; Alanine − 0.594), ketone body metabolism (Acetone − 0.64; 3-Hydroxybutyric Acid − 0.632; Acetonacetic Acid − 0.586), fatty acid (carnitine − 0.509) and carbohydrate metabolism ( Maltose − 0.510; Citric Acid − 0.485). 2–3 Butanediol, a by-product of sugar-fermenting microbial metabolism also decreased (− 0.489). No correlation was seen with change in quadriceps muscle mass for any of the 20 metabolites varying with time (all *p* > 0.05). Increasing severity of organ failure was related to increasing ketone body metabolism (3 Hydroxybutyric Acid-1 and − 3; *p* = 0.056 and *p* = 0.014), carnitine deficiency (*p* = 0.002) and alanine abundancy (*p* − 0.005). A 6-times a day intermittent feeding regimen did not alter metabolite patterns across time compared to continuous feeding in critically ill patients, either within a 24 h period or across 10 days of intervention. Future research on intermittent feeding regimens should focus on clinical process benefits, or extended gut rest and fasting.

## Introduction

Intermittent or bolus enteral feeding regimens in critically ill patients have been of increasing interest to clinicians and scientists^1^. The alternative, continuous feeding, is associated with a variety of physiological and metabolic effects that may be detrimental to patients.

Critically ill patients lose 2–3% of muscle mass each day as a result of decreased muscle protein synthesis and unchecked protein breakdown^2,3^. In healthy subjects, sustained elevated circulating amino acid concentrations due to continuous feeding results in the “muscle full effect”, whereby the protein synthetic response becomes refractory after about 90 min, despite availability of amino acids^4^. In our recent multi-centre single blinded randomised controlled trial, no difference in muscle mass loss was identified between critically ill patients in multi-organ failure randomised to continuous enteral feeding, when compared to those receiving an intermittent feeding regimen^5^.

However, it has been speculated that other less well-explored benefits of intermittent feeding might improve patient outcome or experience^6^. Continuous feeding may contribute to circadian rhythm misalignment, affecting not only metabolism^7^, but also sleep quality (and hence susceptibility to delirium, pain and detrimental cognitive outcomes; and engagement with rehabilitation^8^. Continuous feeding attenuates both Ghrelin and peptide YY diurnal variation with pleiotropic metabolic effects^9^, and increases insulin resistance via inhibition of glucose transport^5^ while also impacting important cellular processes (e.g. inhibiting autophagy which leads to an accumulation of end-products of cellular damage, potentially impeding overall patient recovery^10^).

We hypothesise that intermittent enteral feeding may modulate amino acid, fat and carbohydrate metabolite profiles over time, when compared to continuous feeding. For example, alterations in amino acid trafficking may be advantageous as a result of addressing the muscle full effect, providing support for future trials combining intermittent feeding with a co-intervention addressing bioenergetics failure to improve physical function^4,5^. Decreasing nocturnal carbohydrate and lipid delivery may improve circadian rhythm quality by reducing the dissociation between central (e.g. suprachiasmatic nuclei) timing mechanisms and nutritional stimuli^11,12^. Time epochs without nutrition delivery promote autophagy, advantageously removing cellular debris (e.g. from myonecrosis) which would be reflected in amino acid metabolite abundance^13,14^. We sought to charcterise these changes in plasma metabolite profile through a Nuclear Magnetic Resonance (NMR) based analysis in order to inform the design of future nutritional trials in this population^15^.

## Methods

### Study design and participants

The Intermittent versus Continuous Feeding in critically ill patients study (IVC study, NCT02358512) was a multicentre single-blinded randomised controlled trial performed on 8 UK intensive care units (ICU), in which adult (≥ 18 years of age) patients were randomised (1:1 ratio, concealed allocation) to receive either a continuous or intermittent enteral feeding regimen5.Chest. 158:183–194. The original study received ethics committee approval (National Research Ethics Service Committee London-Queens Square; REC reference 14/LO/1792; IRAS project ID 160,281) and was registered on clinicaltrials.gov before randomisation was commenced (05/11/15). All research was performed in accordance with the Declaration of Helsinki. Prospective informed assent was obtained in writing from a nominated personal consultee or professional consultee. Retrospective participant consent was obtained on return of participant’s mental capacity. Permission to use participants’ data if capacity did not return or they did not survive was included in the assent process.

### A pre-planned targeted metabolomic cohort ancillary study was performed

In brief, patients were required to have been admitted to the ICU for ≤ 24 h prior to enrolment. Included patients were those anticipated to be mechanically ventilated for > 48 h, who required enteral feeding via a nasogastric tube, had multi-organ failure (Sequential Organ Failure Assessment (SOFA) score > 2 in ≥ 2 domains at admission), had a likely ICU stay of > 7 days, and who were considered likely to survive > 10 days. The characteristics and demographics of patients randomised to each limb were well balanced.

### Study procedures

On recruitment, patient age, weight, medical history and APACHE II and SOFA score on admission to ICU, were recorded. Daily nutritional and biochemical data for the 10 day intervention (including volume of enteral feed received, energy and protein targets, urea and creatinine levels), and requirement for renal replacement therapy, were documented.

*Exposure:* The intermittent feeding regimen (the intervention) consisted of six feeds per 24 h, each administered over 3 to 5 min via nasogastric tube.

*Comparator*: The continuous (standard) feeding regimen was delivered continuously over 24 h, as per local guidelines.

More details on the feeding regimens can be found in the original publication and supplement^5^.

### Sample collection

Blood samples were collected in heparinised tubes and the supernatant was removed and frozen at − 80 ^O^C post centrifugation. This occurred twice a day, at the 9 a.m. and 1 p.m. feeds in the intermittent arm. In the continuous feeding arm, blood samples were taken 30 min apart at 9 a.m. and 1 p.m.. These samples were collected as part of the original trial^5^. Samples were collected on days 1 7 and 10, based around our previous observational study^2^. However manpower constraints prevented operationalisation of day 3 sampling.

### Measures of muscle mass

Rectus Femoris Cross-Sectional Area (RFCSA) was measured at randomisation (as baseline) and at day 10 (primary outcome of the original trial)^5^. RFCSA is a validated measure that is increasingly used to measure muscle mass in the critically ill^2,16,17^.

### Metabolite analysis

Blood samples were analysed using a targeted metabolomics approach using Nuclear Magnetic Resonance Spectroscopy (NMR). For each sample, 300 μL of plasma was mixed with 300 μL of a 90/10 H_2_O/D_2_O phosphate buffer at pH 7.4, containing 0.4 mM of NaN_3_ and 0.03 mM TSP, and transferred to 5 mm NMR tubes. NMR experiments were recorded on a Bruker 600 MHz NMR spectrometer equipped with a Prodigy cryoprobe. On each sample, a T_2_-filtered ^1^H NMR spectrum and a diffusion-filtered ^1^H NMR spectrum were acquired, to analyse small molecules and lipoproteins separately, respectively. The T_2_-filtered spectrum was acquired using the PROJECT pulse sequence^18^, with 4 dummy scans, 64 scans, a spectral width of 20.82 ppm, an inter-scan delay of 4 s and an acquisition time of 2.62 s. The diffusion-filtered spectrum was acquired with a pulse sequence using stimulated echoes and LED, along with bipolar gradients and spoil gradients^19^, with 4 dummy scans, 64 scans, a spectral width of 20.82 ppm, a diffusion time of 120 ms, a gradient length of 1.375 ms for the bipolar gradients, an inter-scan delay of 4 s and an acquisition time of 2.62 s. Each spectra was referenced using the TSP peak at 0.00 ppm.

### 2D NMR spectra for metabolites identification

For metabolite identification, 2D NMR spectra were acquired on one of the samples, in particular [^1^H, ^1^H]-TOCSY and [^1^H,^13^C] HSQC. For the [^1^H,^1^H]-TOCSY, the Bruker pulse sequence “dipsi2gpphzs” was used, slightly modified to include presaturation, with 16 scans, 512 t1 increments, a spectral width of 13.7 ppm in both dimensions and a relaxation delay of 2 s. The [^1^H,^13^C]-HSQC spectrum was acquired using the Bruker pulse sequence “hsqcetgpsisp2.2”, with 32 scans, 512 t1 increments, a spectral width of 210 ppm and 20.8 ppm in the ^13^C and ^1^H dimension respectively, and a relaxation time of 2 s. Once acquired, libraries including HMDB^20^ and BMRB^21^ were used to annotate the 2D spectra and, by extension, the 1D spectra.

### Sample size estimate

This was an exploratory study of the metabolome under different feeding regimes in critically ill patients. Without pilot data existing power calculation packages e.g. Metaboanalyst ((https://www.metaboanalyst.ca) are unable to estimate the effect size distribution, the average power and the minimal sample size^22,23^. These 75 subjects represent those patients in whom sampling was performed, as opposed to a pre-specified sample size.

### Statistical analysis

The statistical analyses were performed using R version 4.0.3 (https://cran.r-project.org/) and R Studio (version 1.3.1093). All R scripts were written in house and can be accessed at https://doi.org/10.5281/zenodo.10055412.

Following NMR analyses, separate data matrices of metabolite abundances for lipid species, small molecule species and amino acids were generated and each analysed separately as targeted panels of metabolites. Each metabolite panel was first filtered to remove missing data points and/or those with negative abundance values, followed by log transformation, centering and scaling to bring metabolite abundances within the same dynamic range. Exploratory visualisations were then generated using principal component analyses to assess for any obvious effects of the feeding regimens on metabolite abundances. To determine differences in metabolite abundance between feeding regimens, across time and interactions between feed and time, analyses were performed using a moderated empirical Bayesian mixed effects model approach with the limma (version 3.18) R package, with the primary outcome being a difference in metabolite profiles between arms^24^. This approach ‘borrows’ information on variance in the data to moderate t-statistics in individual comparisons and is more conservative than unmoderated t-tests. Where significant main effects were observed, these were followed up utilising specific contrasts to determine where these effects were being observed within the experimental model. For each comparison, false discovery rate (FDR) correction was applied using the Benjamini–Hochberg approach (q value threshold of 0.05). Significant metabolites were then assessed for their relationship to clinical outcome measures; change in rectus femoris muscle cross sectional area (CSA) and admission Sequencial Organ Failure Assessment (SOFA) Score, where data were available for all patients. Simple linear regression was performed between the significant scaled and transformed metabolite abundances and clinical endpoints to investigate associations. All graphical representations were generated using ggplot2 (version 3.4) from the tidyverse^25^.

Linear mixed effect modelling was chosen over standard statistical approaches (e.g. repeated measures ANOVA) as it deals better with designs where the repeated measure is continuous (i.e. in this case, time), where the design may become unbalanced (e.g. due to attrition) and where non-independence of data due to clustering within individuals exists, as is likely to be the case here. In addition we used limma to generate empirical Bayes estimates of metabolite-wise variance^26^. These variance estimates are shrunk towards a common variance estimate generated by ‘borrowing information’ from all metabolites. This is a form of regularisation in the model fitting and is conservative in the context of small numbers^27^.

### Ethical approval

The study received ethics committee approval (National Research Ethics Service Committee London-Queens Square; REC reference 14/LO/1792; IRAS project ID 160,281) and was registered on clinicaltrials.gov before randomisation was commenced.

## Results

Of the 121 patients randomised, 62 were in the intervention feeding (IF) group, and 59 in the control feeding (CF) group. Five hundred and ninety four samples were available for 75 patients (Table 1), with sampling timepoint breakdown available in Table S1.

**Table 1:** Characteristics of the 75 patients whose metabolome abundances are described. LOS = Length of Stay; APACHE II = Acute Physiology and Chronic Health Evaluation Score; SOFA = Sequentional Organ Failure Assessment; RRT = Renal Replacement Therapy; NMBA = Neuromuscular Blockade administration. Data are mean (95% confidence intervals), except for # indicating median with range.

	Intermittent Feeding (n = 40)	Continuous Feeding (n = 35)
Age, y	55(50.0–60.0)	61 (55–67)
Male, No. (%) ¥	11 (27.5)	9 (25.7)
LOS prior to ICU admission, d ^#^	0.0 (0–15)	0.0 (0–13)
Period ventilated, d ^#^	11 (2–48)	8 (2–41)
ICU LOS, d^#^	16 (3–93)	17 (3–52)
Hospital LOS, d^#^	27 (4–183)	32 (4–102)
APACHE II score	21 (19–24)	20 (17–22)
SOFA score on admission	10 (9–11)	11 (10–12)
ICU survival, No. (%)	28 (70)	29 (83)
Hospital survival, No. (%)	21 (52.5)	28 (80)
RRT, No. (%)	16 (40)	12 (34)
NMBA use, d^#^	0 (0–8)	1 (0–6)
Hydrocortisone dose, mg total by day 10	0 (0–2700)	0 (0–3200)
Gastro-protection, d^#^	9 (0–11)	10 (0–11)
Vasopressors support, d^#^	4 (0–11)	5 (0–22)
Sedation use, d^#^	7 (2–11)	6 (0–11)
Propofol dose by day 10, g	21.1 (15.2–27.1)	23.0 (16.4–29.5)
Admission diagnosis, No. (%)		
Sepsis	13 (32.5)	14 (40)
Cardiogenic shock	9 (22.5)	8 (22.9)
Trauma	5 (12.5)	7 (20.0)
Respiratory failure	5 (12.5)	1 (2.9)
Intracranial haemorrhage	2 (5.0)	1 (2.9)
Acute liver failure	2 (5.0)	2 (5.7)
Acute kidney Injury	2 (5.0)	1 (2.9)
Drug overdose	1 (2.5)	0 (0.0)
Emergency surgery	1 (2.5)	1 (2.9)
Cerebrovascular accident	0 (0.0)	0 (0.0)
Comorbidities, No. (%)		
Hypertension	9 (22.5)	4 (11.4)
Chronic respiratory diseases	9 (22.5)	11 (31.4)
Diabetes Mellitus	8(20.0)	7 (20.0)
Ischemic heart disease	8 (20.0)	10 (28.6)
Psychiatric diseases	6 (15.0)	5(14.3)
Renal impairment	2 (5.0)	5 (14.3)
Obesity	4 (10.0)	1 (2.9)
Liver cirrhosis	3 (7.5)	3 (8.6)
Haem-oncological disease	3 (7.5)	1 (2.9)
Thyroid disease	2 (5.0)	0 (0.0)
Crohn’s disease	0 (0.0)	1 (2.9)
Previous CVA	1 (2.5)	1 (2.9)
Chronic Pancreatitis	1 (2.5)	0 (0.0)

Plasma metabolomic analyses using NMR identified a total of twenty-four amino acid metabolite features, nineteen lipid based metabolite features and forty-four other small molecule metabolite features across five hundred and ninety observations/samples (Figs. S1 and S2). Four samples were removed from the final analyses due to missing data points, or poor quality data output (e.g. negative abundance). Each of these individual metabolite panels were then taken forward for further analyses.

### Exploratory visualisations

**Figure 1:**
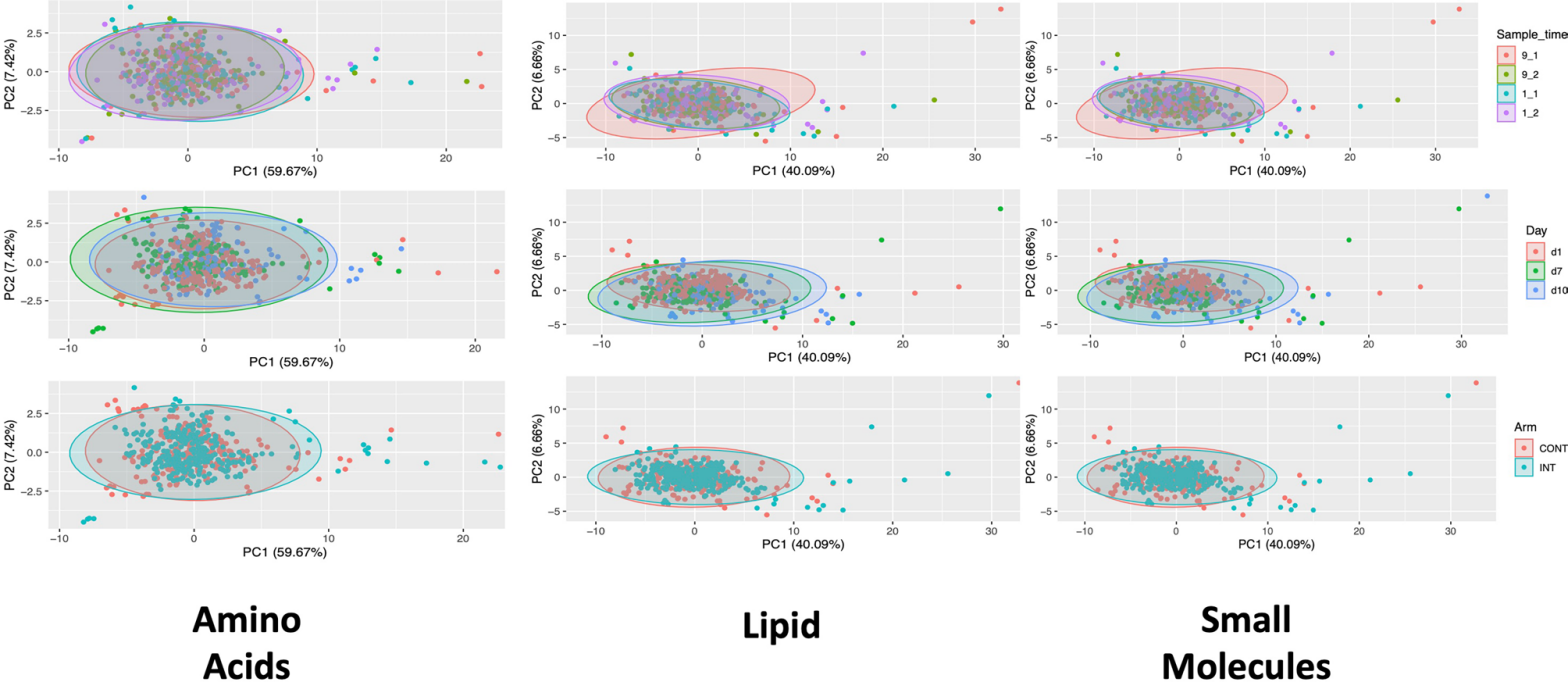
(**A**–**C**) Principal Component Analyses of; (**A**) Amino Acid Metabolites, (**B**) Lipid Metabolites and (**C**) Small Molecule Metabolites to assess for patterns of variability that might underpin physiological changes due to time of day (top graph in panel), time since admission (middle graph in panel) and feeding regimen (bottom graph in each panel).

We first examined the metabolite panels for broad patterns of variability that might underpin physiologically relevant changes as a result of feeding regimen; time since admission or time of day. Although a few observations from each panel were clustering out with others, along the main two axes of variation (PC1—explaining between 40 and 60% of variance and PC2—explaining 6 and 8% of variance) no broad patterns distinguished between intermittent or continuous feeding arms, across intra-day sampling times or over the 10 days from initial ICU admission (Fig. 1A–C).

### Specific metabolite alterations

**Figure 2:**
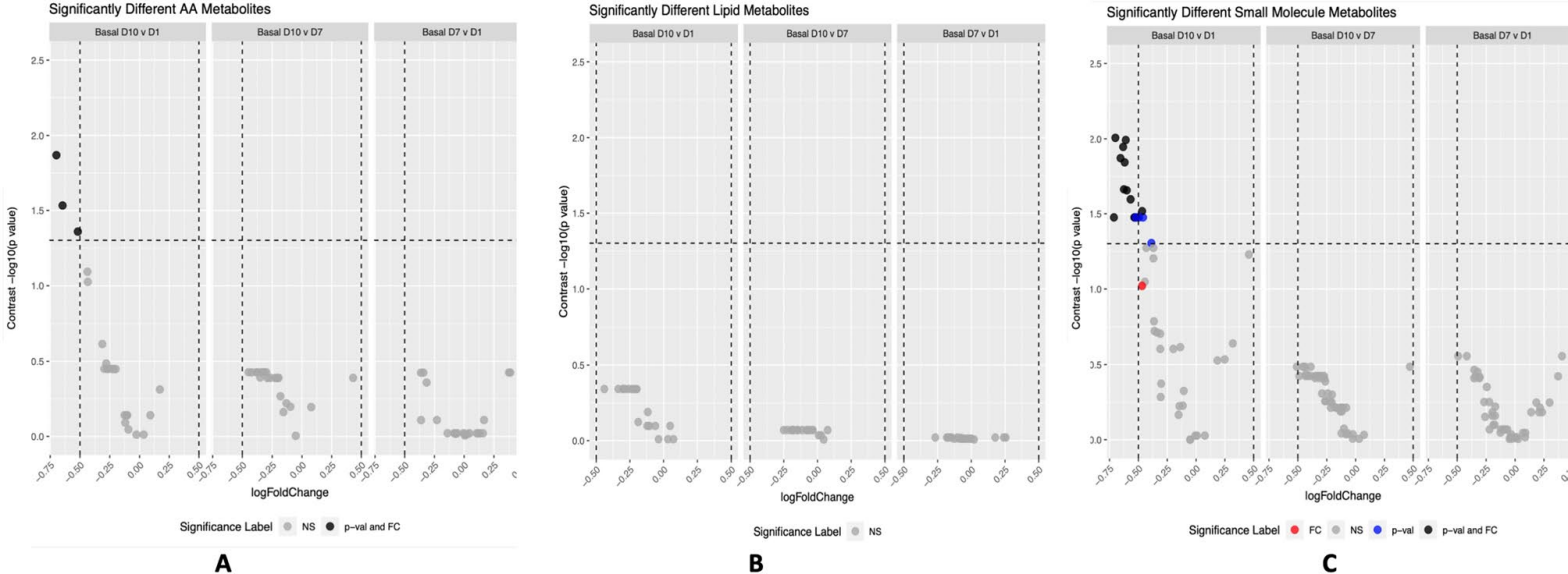
(**A**–**C**) Volcano Plots depicting numbers of metabolites whose abundance altered over time, independent of feeding regimen across length of ICU admission for; (**A**) Amino Acid Metabolites, (**B**) Small Molecule Metabolites and (**C**) Lipid Metabolites. Vertical and horizontal cutoffs represent -log10 False Discovery Rate corrected p value and log fold change respectively, with differences from day 10 to day 1 presented in the left hand panel, differences from day 10 to day 7 in the middle panel and differences from day 7 to day 1 in the right hand panel. Four possible outcomes are presented; NS = Non-significant, FC =  > than log fold change cut off (0.5) ; *p*-val = FDR corrected significant change and *p*-val and FC = FDR corrected significant change and > log fold change cut off.

We next addressed the question of specific metabolite alterations between the feeding arms using a mixed effect moderated empirical Bayesian approach^24^. Comparison of feeding arms revealed no statistically significant abundance differences after FDR correction for multiple testing (data not shown) between arms at individual sampling times. Data were therefore collapsed across feeding arms and sampling times and examined for an effect of duration of ICU stay using the first sample collected on each day. After FDR correction for multiple testing no lipid based molecules, and only two amino acids (glutamine and alanine), decreased in abundance over the 0–10 day period. Several other small molecules (see Table 2) also decreased in abundance over this period, with no variation seen across the earlier time points (Fig. 2A–C and Supplementary Fig. S3). Putative identification of these metabolites showed a signature related to metabolites involved with ketone body metabolism, energy/nutrient metabolism and amino acid metabolism (Table 2).

**Table 2:** Summary of all metabolites and associated Human Metabolite Database (HMDB) ID number and related biological pathway whose concentration was found to change significantly over the duration of ICU stay, following removal of a non-significant feeding arm effect by collapsing data into a single dataset together with their associated Human Metabolite Database (HMDB) ID number and related biological pathway. Where two peaks of the same metabolites are seen (representing different isomers) these are numbered separately. “Unknown” refers to metabolites not seen in the HMDB.

Metabolite	log fold change abundance	t Statistic	*p* value	FDR Corrected *p* value	HMDB number	Metabolic pathway
Dimethylsulfone	− 0.6842	− 3.7749	0.0002	0.0099	HMDB0004983	Sulphur Metabolism
Acetone	− 0.6369	− 3.5741	0.0005	0.0102	HMDB0001659	Ketone Body Metabolism
Lactic acid	− 0.6338	− 3.4274	0.0008	0.0113	HMDB0000190	Gluconeogenesis/Pyruvate Metabolism
3-Hydroxybutyric Acid 2	− 0.6322	− 3.2916	0.0012	0.0134	HMDB0000442	Ketone Body Metabolism
3-Hydroxybutyric Acid 1	− 0.6082	− 3.2042	0.0016	0.0143	HMDB0000442	Ketone Body Metabolism
Acetonacetic Acid	− 0.5863	3.0174	0.0030	0.0217	HMDB0304256	Ketone Body Metabolism
Unknown 6	− 0.5750	− 2.9628	0.0035	0.0220	Unknown	Unknown
Carnitine	− 0.5087	− 2.8732	0.0046	0.0253	HMDB0000062	Fatty Acid metabolism
Pyroglutamic acid	− 0.5254	− 2.7728	0.0062	0.0303	HMDB0000267	Amino Acid Metabolism
Maltose	− 0.5096	− 2.6715	0.0083	0.0334	HMDB0000163	Starch and Sucrose Metabolism
Myo-inositol	− 0.4809	− 2.6043	0.0101	0.0334	HMDB0000211	Galactose/Inositol Metabolism
Pyridoxine	− 0.6603	− 2.5944	0.0103	0.0334	HMDB0000239	Vitamin B6 Metabolism
3,4-Dihydroxybenzoic acid	− 0.4918	− 2.5793	0.0108	0.0334	HMDB0001856	Hydroxybenzoic Acid Derivatives
Citric Acid 2	− 0.4851	− 2.5469	0.0118	0.0334	HMDB0000094	Energy Metabolism/TCA Cycle
2,3-Butandiol	− 0.4888	− 2.5452	0.0119	0.0334	HMDB0003156	Microbiome Metabolism
Citric Acid 4	− 0.5101	− 2.5361	0.0122	0.0334	HMDB0000094	Energy Metabolism/TCA Cycle
Methanol	− 0.4468	− 2.3665	0.0191	0.0495	HMDB0001875	Primary Alcohol Metabolism
Glutamine	− 0.6828	− 3.4989	0.0006	0.0135	HMDB0000641	Amino Acid Metabolism
Alanine 2	− 0.5938	− 3.0605	0.0025	0.0293	HMDB0000161	Amino Acid Metabolism
Alanine	− 0.5407	− 2.7981	0.0057	0.0437	HMDB0000162	Amino Acid Metabolism

### Clinical correlates

Finally, to assess whether there was any relationship between these groups of metabolites and clinical outcome following ICU admission, we used linear regression analysis to examine the relationship between the 20 metabolites varying over time and the primary clinical endpoint of change in muscle mass during admission (restricted to only n = 35 for this analyses due to need for complete data sets for all at 0 and 10 days). Patients in this cohort had a mean RF_CSA_ of 455.4 cm^2^ (95%CI 386.3–524.6) on admission and lost 17.6% (95%CI − 22.1% to − 14.2%) of muscle mass over the 10 day study period. No significant relationship was found (all *p* > 0.05; Table 3 and supplementary data Fig. S4). Weak relationships were seen between baseline abundance of significantly changing metabolites and organ failure scoring (see Table 4 and supplementary Fig. S5).

**Table 3:** Summary of Linear Regression Analyses of relationship between significantly changing metabolites and change in muscle cross sectional area over 10 day ICU stay. Where two peaks of the same metabolites are seen (representing different isomers) these are numbered separately. “Unknown” refers to metabolites not seen in the HMDB.

Metabolite	R Squared	*p* value
Acetone	0.0032	0.7520
Acetonacetic acid	0.0691	0.1331
3-Hydroxybutyric	0.0418	0.2461
3-Hydroxybutyric	0.0582	0.1692
Unknown 6	0.0381	0.2684
Citric acid 2	0.0007	0.8849
Citric acid 4	0.0276	0.3296
Dimethylsulfone	0.0357	0.3479
Carnitine	0.0032	0.2846
Methanol	0.0263	0.3591
2,3-Butandiol	0.0297	0.3294
Maltose	0.0157	0.4803
Myo-inositol	0.0691	0.1330
Lactic acid	0.0060	0.6645
Pyroglutamic acid	0.0234	0.3876
3,4-Dihydroxybenzoic acid	0.0810	0.1029
Pyridoxine	0.0449	0.2291
Alanine	0.0006	0.8891
Glutamine	0.0176	0.4542
Alanine 2	0.0000	0.9853

**Table 4:** Summary of Linear Regression Analyses of relationship between significantly changing metabolites and Sequential Organ Failure Assessment (SOFA) score over a 10 day ICU stay. Where two peaks of the same metabolites are seen (representing different isomers) these are numbered separately. “Unknown” refers to metabolites not seein the HMDB.

Metabolite	R Squared	*p* value
Acetone	0.0077	0.4772
Acetonacetic acid	0.0173	0.2848
3-Hydroxybutyric acid 1	0.0541	0.0562
3-Hydroxybutyric acid 2	0.0875	0.0143
Unknown 6	0.0507	0.0648
Citric acid 2	0.0071	0.4949
Citric acid 4	0.0048	0.5760
Dimethylsulfone	0.0027	0.6735
Carnitine	0.1397	0.0017
Methanol	0.0028	0.6682
2,3-Butandiol	0.0038	0.6176
maltose	0.0005	0.8617
Myo-inositol	0.0370	0.1161
Lactic acid	0.2018	0.0001
Pyroglutamic acid	0.0127	0.3609
3,4-Dihydroxybenzoic acid	0.0002	0.9076
pyridoxine	0.0746	0.0243
Alanine	0.1131	0.0050
Glutamine	0.0524	0.0604
Alanine 2	0.0067	0.5056

## Discussion

We performed a detailed metabolic analysis of amino acid, lipid and small molecule profiling in response to intermittent enteral feeding compared to continuous enteral feeding in critically ill patients, in the context of a multi-centre randomised controlled trial. We examined these three specific metabolite panels, composed of twenty four amino acid metabolites, nineteen lipid based metabolites and 44 small molecule metabolites over a four hour window within a day and longitudinally up to 10 days, in addition to between arms. No differences were seen between groups at each of the individual time points. Using a mixed effect moderated empirical Bayesian approach, we confirmed this finding, allowing us to collapse the study arms and examine changes over time of specific metabolites. Decreases in relative concentrations of small metabolites related to fatty acid, ketone acid and carbohydrate metabolism were observed over the first 10 days of critical illness.

Metabolic profiling of these critically ill patients was surprisingly stable over time, with alterations only being seen at 10 days as opposed to variations at earlier timepoints. This may reflect the population chosen, as all patients had multi-organ failure, and reach an abnormal equilibrium of cellular metabolism early, and resolve slowly. This is in keeping with our previous observations in skeletal muscle and the limited clinical metabolomics data in critically ill patients^2,28,29^*.* These findings were also suprising given the differential recovery of insulin sensitivity between trial arms between day 7 and day 10^5^. While metabolite profiling may discriminate between healthy subjects and patients with insulin resistance in stable settings, insulin resistance is a dynamic phenomenon in the critically ill, and may have attenuated effects on metabolite abundance^30^.

Decreases in circulating glutamine have been well described in the critical care literature, although supplementation is associated with an increase in mortality, perhaps through ammonia generation^31–33^. Reductions in plasma Alanine concentration may reflect an increased throughput of the Cahill cycle to generate Adenosine Tri-Phosphate, in an attempt to redress cellular bioenergetic failure in critical illness^29,34^. Beta-oxidation is impaired in the skeletal muscle critically ill patients, and this may be reflected in the unchanged lipid metabolite concentrations over time, though the degree to whicih appendicular muscles contribute to total energy expenditure in a resting and sedated ICU patient is unclear ^29^. Decreases in circulating carnitine likely reflect the decrease in intramuscular carnitine pallmitoyltransferase concentrations in critical illness^29^. Decreases in carbohydrate metabolites likely reflect impaired oxidative phosphorylation which, in combination with impaired beta-oxidation, may result in substrate switching and corresponding ketone body metabolism^28,35,36^. Increasing severity of organ failure was related to increasing ketone body metabolism, carnitine deficiency and alanine abundance in keeping with the processes described above. The relationship between increasing lactate and illness severity is well described.

2,3-Butandiol is produced from sugar-fermenting microbes (e.g. Klebsiella sp, Entrobacter Sp, Serratia Sp, Bacillus Sp), and the decrease in metabolite levels are likely to reflect dysbiosis of critical illness, of which our understanding is still nascent^37,38^.

Teasing apart the relationship between these processes across time and determining the true biological significance of the findings remains challenging, and will remain so until experimental and interventional studies can perturb these processes in the critically ill. Our data are the first to demonstrate these changes longitudinally as opposed to across cohorts, strengthening the case for focusing intervention development on these pathways. Metabolite profiling has been used in the past to determine effects of interventions such as Vitamin D^39^. In this publication, we separated metabolites into three categories- amino acids, lipids and small molecules, refining metabolite signatures in critical illness. These refinements may be useful in standardising comparisons in metabolite profiling within randomised controlled trials. The ability to compare data across trials is fundamental to evidence synthesis and field development, and as metabolite profiling becomes more commonplace, such standardisations will increase in importance.

We were unable to demonstrate a difference in metabolite patterns between trial arms at individual timepoints. These data suggest that intermittent feeding is unable to sufficiently alter metabolism in critically ill patients, and therefore is unlikely to be better than continuous feeding in affecting end-organ metabolism. This does not, however, rule out the existence of other benefits of intermittent feeding regimens, such as increased nutrition delivery by overcoming difficulties in the process of nutritional delivery to the critically ill patient^5,40^. Intermittent or bolus feeding regimes have not been standardised, and there remains debate on the methodology, and comprehensive reviews exist(ref). The effect on the metabolic outcomes of selecting a different intermittent feeding regimen is unknown Other untested benefits of intermittent feeding regimens include beneficial impacts on autophagy, which periods of intermittent fasting may promote^41,42^. Testing these two hypotheses requires further prospective randomised trials, as does studying the effect of intermittent feeding on circadian rhythms and sleep quality^43,44^. Lastly, the role of coordinated intermittent feeding and exercise regimens needs to be tested in the recovery phase of critical illness, as the two processes are synergistic in stimulation of muscle protein synthesis^45^.

These data represent one of the the largest matrices of metabolomics data in critical illness, as a result of the repeated sampling within days and across 7–10 days which, to our knowledge, has not been performed previously. The multi-centre nature of the trial suggests that these data have significant external validity. Most of the results presented are independent of feeding regimen across length of ICU admission**,** allowing us to use all of the high quality trial biorepository samples to increase study power. This was advantageous as we were unable to pre-determine effect or sample size. These data will inform the design and approach of future studies in critically ill patients, by both enabling a priori statistical plans and a uniform method of handling datasets and longitudinal sampling. The lack of difference in primary outcome measure limited our ability to differentiate reponse/non-response. Muscle metabolite profiling would have been an attractive addition, although this was impractical in the setting of a multi-centre randomised controlled trial. Furthermore, we took advantage of NMR-based metabolic profiling benefits, including non-destructive nature of sample analysis, and the ability to accurately quantitate metabolite levels and to accurately annotate metabolites (an inherent issue for MS) and high level of experimental reproducibility^46^. Analyses were based on 87 metabolites which is not unusual for NMR studies, with a range of 50 to several hundred being usual on most common platforms^46^*.* Future work may benefit from Mass Spectrometry based metabolomics with increased coverage.

## Conclusions

A 6-times per day intermittent feeding regimen did not alter metabolite patterns across time compared to continuous feeding in critically ill patients, within a 24 h period, nor across a 10-day intervention. Future research on intermittent feeding regimens should focus on clinical process benefits, or extended gut rest and fasting.

### Supplementary Information

Supplementary Information.

## Data Availability

Datasets generated are available on reasonable request to the corresponding author (Z.puthucheary@qmul.ac.uk).

## Code availability

Code used for analyses is available at: https://doi.org/10.5281/zenodo.10055412.
