# Supplementary material for: The metabolic effects of intermittent versus continuous feeding in critically ill patients
Source: Sci Rep. 2023 Nov 9;13:19508. doi: 10.1038/s41598-023-46490-5 (PMC10636009; doi:10.1038/s41598-023-46490-5)
Supplement: Supplementary file 1 — Supplementary Information. [file 41598_2023_46490_MOESM1_ESM.pdf]

ONLINE SUPPLEMENTARY DATA

Day	1	1	1	1	7	7	7	7	10	10	10	10
TImepoint	Pre feed 0900	Post feed 0900	Pre feed 1300	Post feed 1300	Pre feed 0900	Post feed 0900	Pre feed 1300	Post feed 1300	Pre feed 0900	Post feed 0900	Pre feed 1300	Post feed 1300
Intermittent feeding	37	38	37	38	21	22	22	22	20	20	18	16
Continuous feeding	32	33	32	31	21	22	19	20	20	18	18	17

Table S1: Sample numbers analysed in the first 10 days. Samples were taken immediately before and 30 minutes after intermittent feeds at 9:00 and 13:00 in the intervention arm and at equivalent timepoints in the control arm

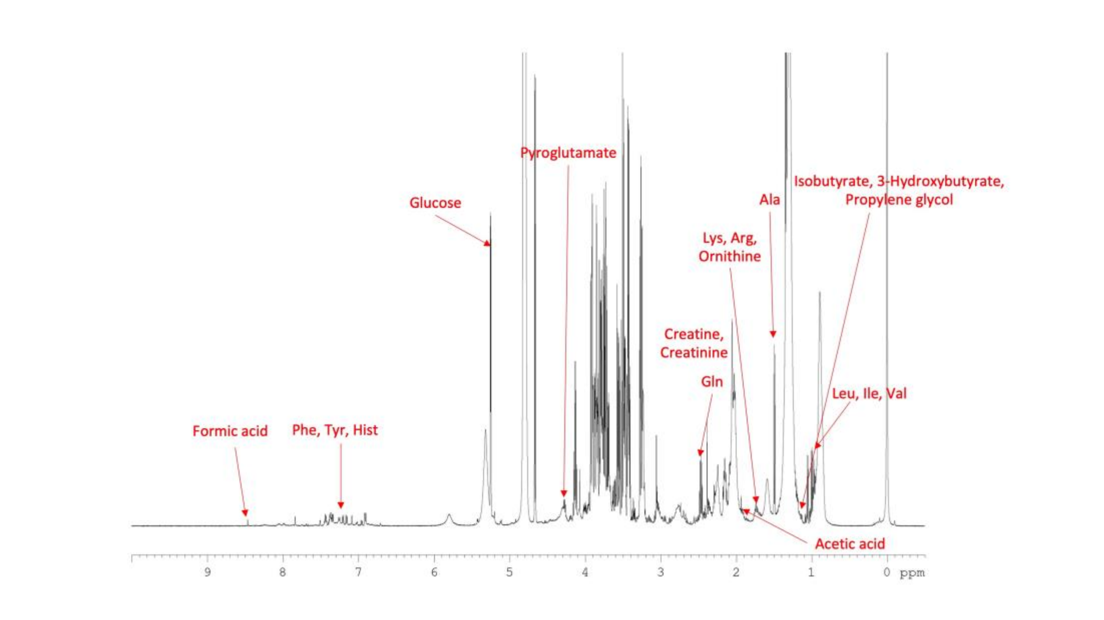

Figure S1: Annotated 1D T_2-edited spectrum of 594 samples recorded on a 600 MHz NMR instrument as detailed in the methods. Assigned metabolites are indicated. Abbreviations: Leu: Leucine, Ile: Isoleucine, Val: Valine, Ala: Alanine, Lys: Lysine, Arg: Arginine, Gln: Glutamine, Phe: Phenylalanine, Tyr: Tyrosine, Hist: Histidine.

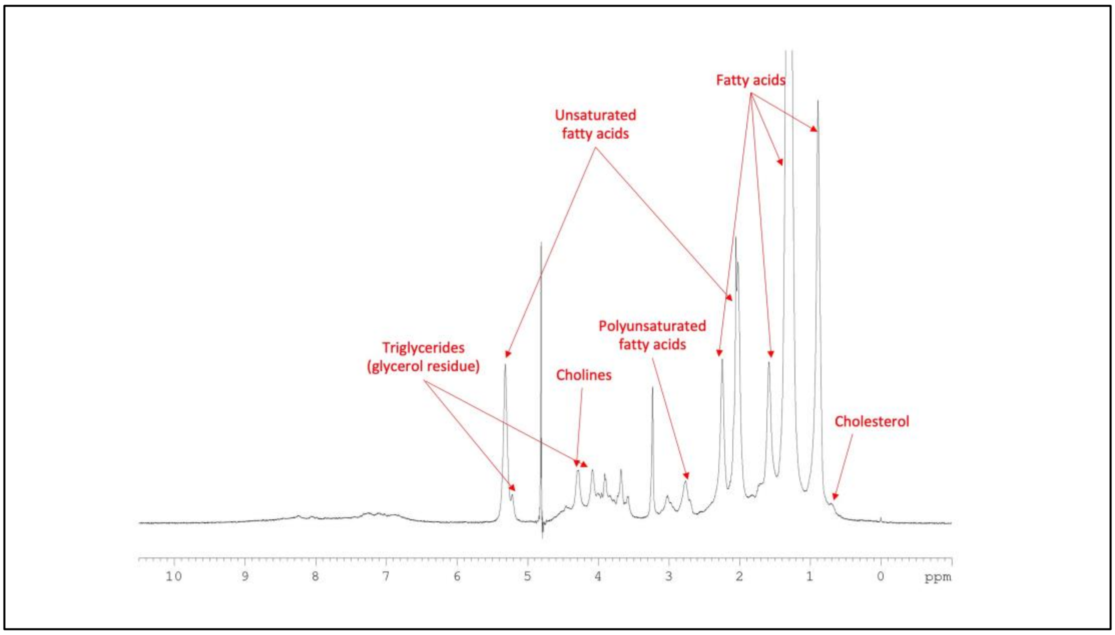

Figure S2: Annotated 1D diffusion-edited spectrum of 594 samples recorded on a 600 MHz NMR instrument as detailed in the methods. Assigned metabolites are indicated.

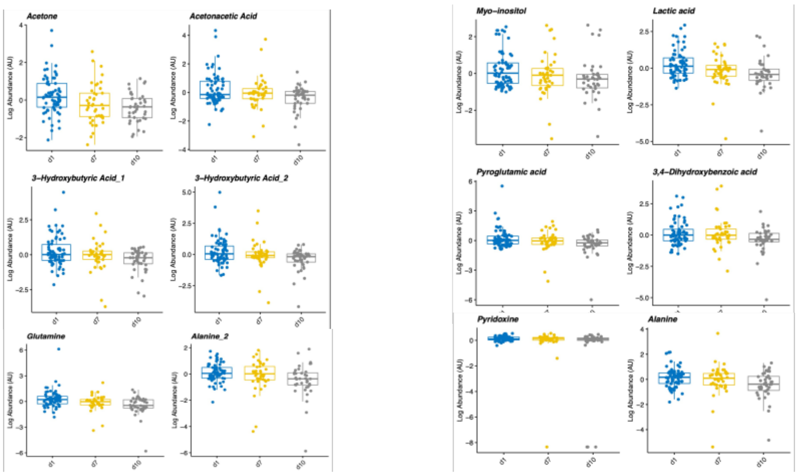

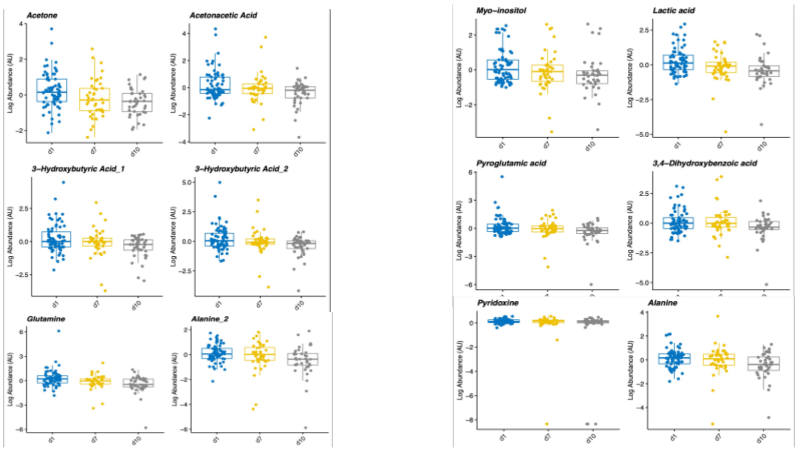

Figure S3. Individual Boxplots Representing Change in Metabolite Abundance over duration of ICU stay for those metabolites showing as statistically significant from mixed effect moderated Bayesian analyses

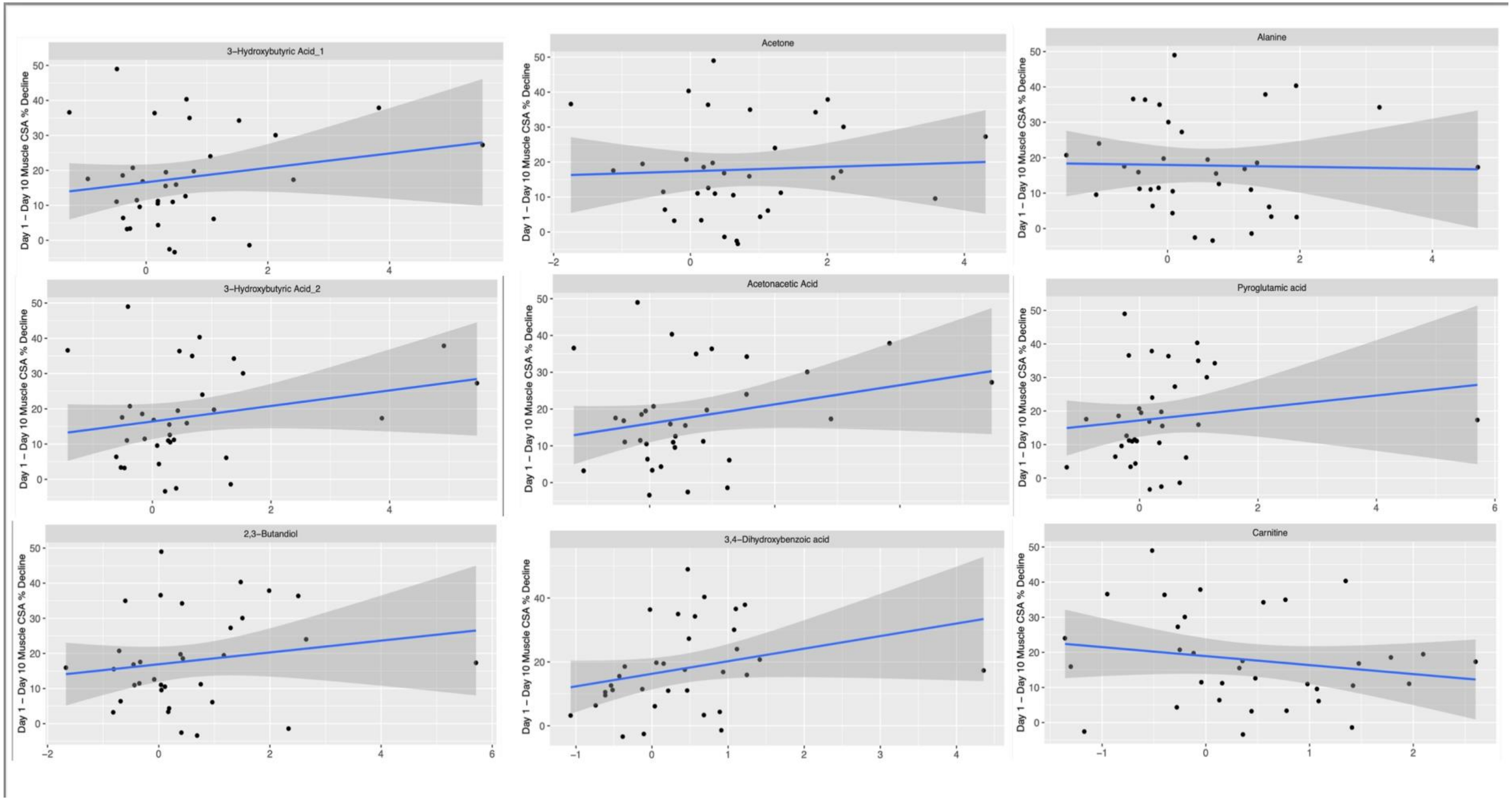
3-Hydroxybutyric Acid_1
Acetone
Alanine
3-Hydroxybutyric Acid_2
Acetonacetic Acid
Pyroglutamic acid
2,3-Butandiol
3,4-Dihydroxybenzoic acid
Carnitine
Day 1 – Day 10 Muscle CSA % Decline

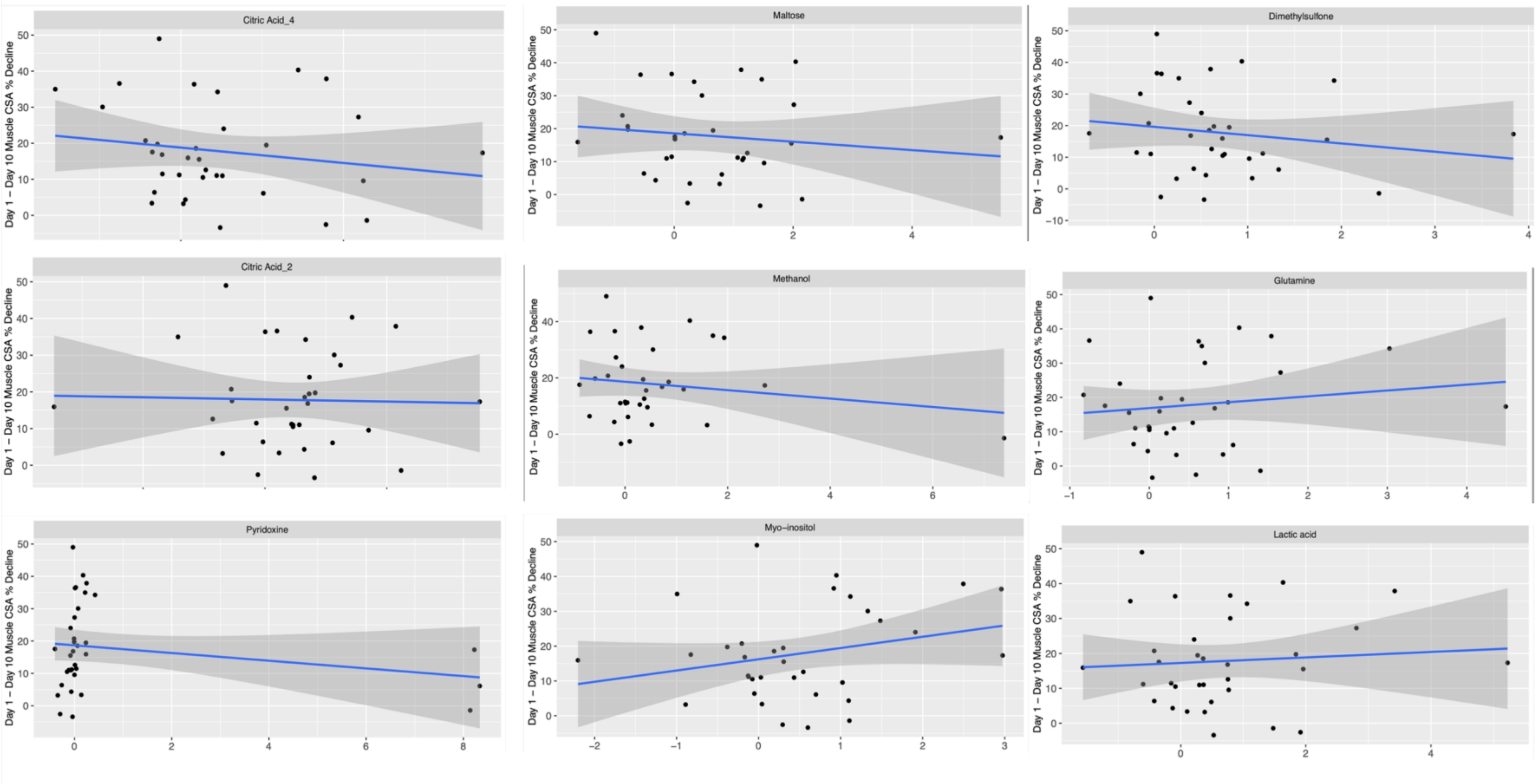

Figure S4. Individual Regression Plots for significant metabolites to assess relationships between change in metabolite abundance due to ICU stay and muscle CSA clinical outcome measure.

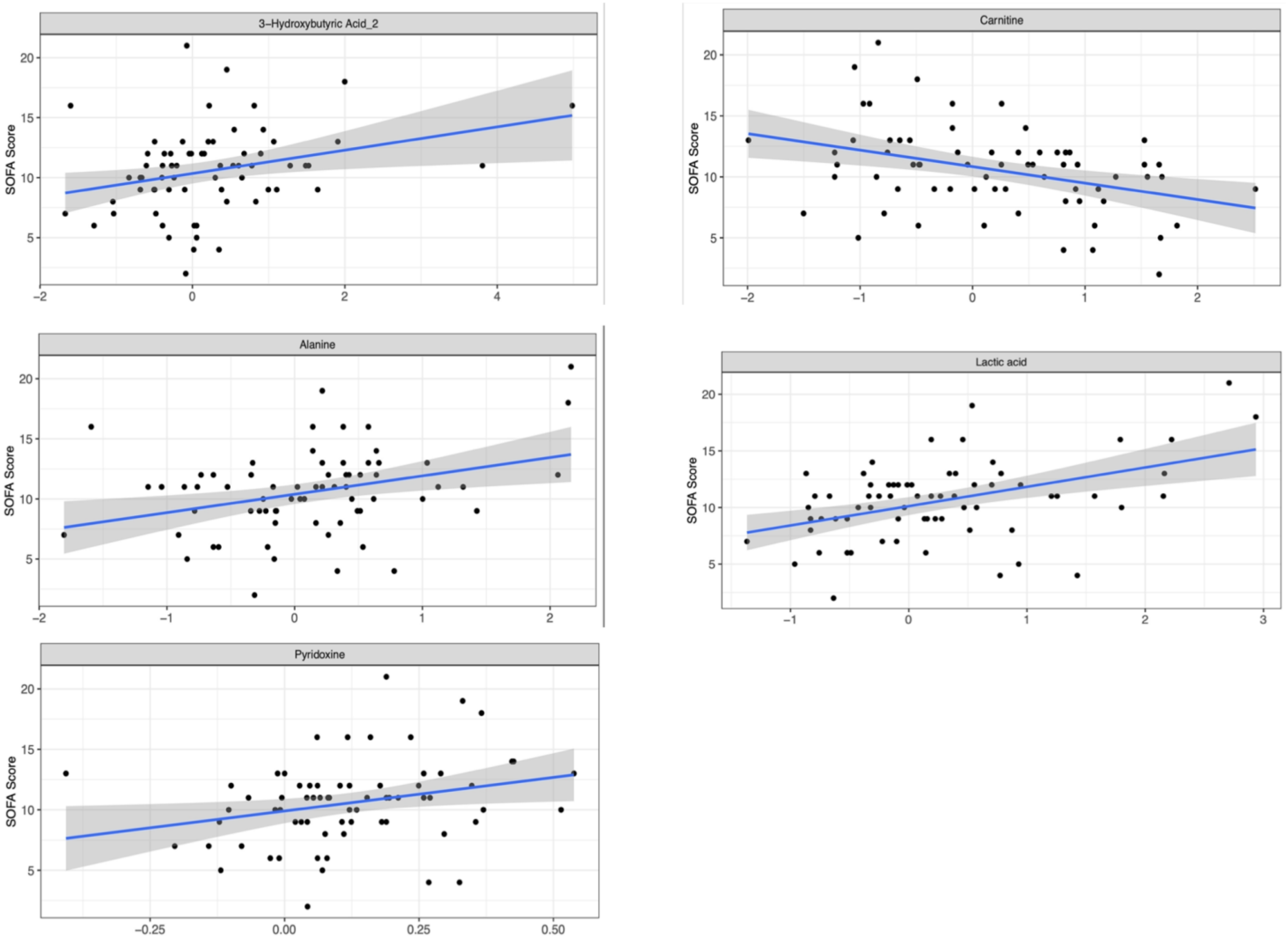

Figure S5: Individual Regression Plots for significant metabolites to assess relationships between change in metabolite abundance due to ICU stay and muscle CSA clinical outcome measure.
